# Will Omics Biotechnologies Save Us from Future Pandemics? Lessons from COVID-19 for Vaccinomics and Adversomics

**DOI:** 10.3390/biomedicines11010052

**Published:** 2022-12-26

**Authors:** Alessandra Ferraresi, Ciro Isidoro

**Affiliations:** Laboratory of Molecular Pathology, Department of Health Sciences, Università del Piemonte Orientale, Via Solaroli 17, 28100 Novara, Italy

**Keywords:** COVID-19, vaccines, omics, personalized vaccination, vaccinomics, adversomics, infectious diseases

## Abstract

The COVID-19 pandemic had cross-cutting impacts on planetary health, quotidian life, and society. Mass vaccination with the current gene-based vaccines has helped control the pandemic but unfortunately it has not shown effectiveness in preventing the spread of the virus. In addition, not all individuals respond to these vaccines, while others develop adverse reactions that cannot be neglected. It is also a fact that some individuals are more susceptible to infection while others develop effective immunization post-infection. We note here that the person-to-person and population variations in vaccine efficacy and side effects have been studied in the field of vaccinomics long before the COVID-19 pandemic. Additionally, the field of adversomics examines the mechanisms of individual differences in the side effects of health interventions. In this review, we discuss the potential of a multi-omics approach for comprehensive profiling of the benefit/risk ratios of vaccines. Vaccinomics and adversomics stand to benefit planetary health and contribute to the prevention of future pandemics in the 21st century by offering precision guidance to clinical trials as well as promoting precision use of vaccines in ways that proactively respond to individual and population differences in their efficacy and safety. This vision of pandemic prevention based on personalized instead of mass vaccination also calls for equity in access to precision vaccines and diagnostics that support a vision and practice of vaccinomics and adversomics in planetary health.

## 1. Health Policy in the Management of COVID-19 and Future Pandemics

The beta-coronavirus SARS-CoV-2, the causative agent of the COVID-19 pandemic, is culpable, often in concert with other morbid or predisposing conditions, for the deaths of more than six million worldwide from November 2019 to the present, and is also responsible for the long-lasting morbidity in various organs of infected patients (so-called “Long COVID”). In addition, the COVID-19 pandemic had enormous adverse impacts on quotidian life in society. Even more significant is that the pandemic laid bare the long-standing social injustices in planetary health, not to mention the organizational shortcomings of health systems and services worldwide. If we gaze back over more than two years of pandemic experiences, the measures have so far included confinement of infected areas (lock-down), quarantine of infected individuals, individual protections and social distancing, and mass vaccination. On the one hand, these measures are justified by the goal of the common good and planetary health, but on the other hand, these measures contest individual rights and freedoms while also failing to acknowledge individual and population differences in vaccine efficacy and safety. The responsibility implicit in such health policy choices seems obvious, and it is thus appropriate to reflect on their effectiveness and on how the use of current medical and biotechnological knowledge could help promote more effective management of the pandemic.

In this regard, it should be stressed that there is a risk associated with taking decisions based on the assumption that something is true just because it has not been proved false due to the lack of knowledge (what is called “argumentum ad ignorantiam”), an alert recently raised by the repurposing of drugs for COVID-19 treatment [1], for example, but that holds for COVID-19 management more generally.

In the context of the massive health emergency imposed by an infectious disease that reached the scale of a pandemic, rapid decisions had to be made in a chaotic climate of uncertainty and lack of complete knowledge. It is acceptable (if not understandable) for a generalized lock-down to be implemented while waiting for a vaccine to become available, and then to proceed to mass vaccination while making every effort possible to respect the principles of democracy and the human rights and agencies of citizens and patients. Reconciling individual and collective good is often one of the key challenges in the critical governance of pandemics.

Pandemics and health emergencies also call attention to fostering science and research cultures that enable systems thinking and long-term vision beyond immediacy.

It is noteworthy that many other infectious diseases are (re)appearing on the planetary health scene, foreshadowing or adding to the present health emergencies [2,3,4]. It is likely that the same pattern of management experienced for COVID-19 might be repeated for these emerging planetary health concerns. In fact, in the view of the authors of this perspective, government experts and advisors appear to be in favor of the way COVID-19 has been and is being dealt with becoming the standard protocol for managing upcoming epidemics and pandemics, under the supervision and command of the World Health Organization. This also calls for rethinking the lessons learned so far from COVID-19.

Thus, what have we learned in these two years, and how can we build on our knowledge of COVID-19 and vaccines to better manage upcoming pandemics as well?

## 2. Omics Studies for Understanding the Immune Response to SARS-CoV-2

Considering that COVID-19 is a novel disease, research on the disease and its short and long-term effects is still emerging [5]. Here we briefly report the omics studies that helped to understand the infection and the development of the disease. From a clinical point of view, COVID-19 presents with a severe acute respiratory syndrome (SARS) that resembles, yet in a milder form and with lower fatality rate, that caused by the two other *Betacoronavirus*, namely SARS-CoV and MERS-CoV [6]. At present, there are a limited number of genomics and epigenomics studies on COVID-19, and it seems that those few that are available have not yet found their way into the fora of the experts on institutional scientific advisory boards who then determine the health management of the pandemic. To infect cells, the virus primarily (but not exclusively) uses angiotensin-converting enzyme 2 (ACE2), which is widely expressed on the membranes of several cell types and tissues [7]. Genetic linkage studies helped to identify ACE2 polymorphisms linked to individual susceptibility to SARS-CoV-2 infection and risk of developing the hyperinflammatory reaction that may result in thromboembolisms, organ failure, and eventually, patient death [8].

The cytotoxic T- and B-cell immune response to SARS-CoV-2 infection follows the recognition of processed viral peptides bound to the MHC class II of antigen-presenting cells. Next-generation sequencing studies have found that certain MHC-II HLA variants and specific HLA haplotypes are associated with individual immune responses to SARS-CoV-2 and with susceptibility to infection and clinical outcome of COVID-19 [9,10,11]. Whole exome sequencing was used to identify rare variants in a set of genes and HLA alleles that could predispose children with COVID-19 to developing multisystem inflammatory syndrome [12].

The identification of HLA MHC-II variants/haplotypes associated with the immune response to SARS-CoV-2 antigens could help to better stratify patients for a personalized therapeutic regimen and vaccination.

## 3. Anti-COVID-19 Vaccines in the Omics Era: One Size Fits All?

Because of the high lethality of the infection and the pressure on the health care systems that oversaw hospitalization in the intensive care unit, vaccine discovery and vaccination have been seen as the primary and most appropriate solution. More recently, however, antiviral drugs and theranostics, which refers to the fusion of therapeutics and diagnostics, have come to the fore for pandemic versus endemic COVID-19 management [13]. The age-old maxim ‘one size medicine does not fit all’ applies not only to antivirals and drugs but also to vaccines and health interventions broadly. This vision in effect calls for a personalized/precision medicine approach to planetary health emergencies.

We live in an extraordinary era in which high-throughput omics biotechnologies have revolutionized the way of identifying the cause of human diseases and infections as seen through the lens of precision/personalized medicine [14]. Let us leave aside the risks of relying totally and uncritically on diagnostic hyper-technology and the implicit risk of hyper-care. Instead, let us see how we can make use of omics biotechnologies to better understand the pathology of COVID-19 and other infections, and how vaccines work in order to optimize their efficacy and safety. A new generation of anti-COVID-19 vaccines have been designed according to criteria dictated by genomics and immunomics and make use of recombinant biotechnologies [15,16]. A systems vaccinology approach was used to profile the signature of the immune response to the BNT162b2 mRNA vaccine [17]. mRNA vaccines have been shown to be able to prevent/attenuate the serious damage of SARS-CoV-2 infection [18], yet their efficacy is unfortunately short-lived, on the order of months rather than years [19,20,21]. More disappointingly, concerns about the safety of these vaccines have been raised especially when considering the subject’s age, sex, ethnicity, previous infection, and other genetic factors [22,23].

It is therefore appropriate to reflect on how the use of current multi-omics biotechnologies could help promote more effective and proactive management of the pandemic, with a view to prevent future planetary health crises as well. The responsibility implicit in such health policy choices for the benefit of public and planetary health seems obvious. This is all with a view to move from one-size-fits-all vaccination, which, as mentioned, is justified in times of emergency, to personalized/precision vaccination, including for the management of endemic COVID-19. Personalized/precision vaccines ought to be made available on a planetary scale, as should all essential medicines and health interventions with critical importance for planetary health.

## 4. Toward Vaccinomics and Adversomics

A vision of precision/personalized vaccines is not in conflict with population vaccination, as such a vision is poised to enhance the efficacy and safety of vaccines. All in all, precision/personalized medicine is a theory for rational therapeutics and prevention that also applies well to vaccines: “the right vaccine, for the right patient, at the right dose, and the right time” [24]. Emerging technologies such as antibody repertoire sequencing, HLA polymorphism genotyping, and high-throughput epitope characterization may assist in designing more effective and safer patient-oriented vaccines [25].

Vaccinomics is concerned with mechanisms of person-to-person and population variations in vaccine efficacy and side effects [26].

Specularly, adversomics makes use of omics and systems biology for investigating the mechanisms of individual differences in the side effects of a given vaccine at genetic and molecular levels [27].

Genomics and systems biology have helped understand the genetic factors that play a role in the adverse effects raised by vaccines [27]. For instance, specific SNPs/haplotypes in the *MTHFR* (methylenetetrahydrofolate reductase, an enzyme involved in homocysteine metabolism) and *IRF1* (interferon regulatory factor-1, which regulates the transcription of IFN type 1 and type 2) genes were significantly associated with systemic adverse events after smallpox vaccination [28]. Immunogenomics has been employed for identifying polymorphisms in cytokine and cytokine receptor genes involved in the immune response to the smallpox vaccine [29].

## 5. State of the Art in Vaccinomics and Adversomics in COVID-19

Much to our disappointment, there has thus far been a delay in the mainstreaming of vaccinomics and adversomics that could make it possible to optimize the efficacy and safety of vaccines for COVID-19 and other infectious pathogens in planetary health. At the same time, there is ongoing hope for a move toward vaccinomics- and adversomics-guided precision/personalized vaccines [25,30,31,32].

**Figure 1:**
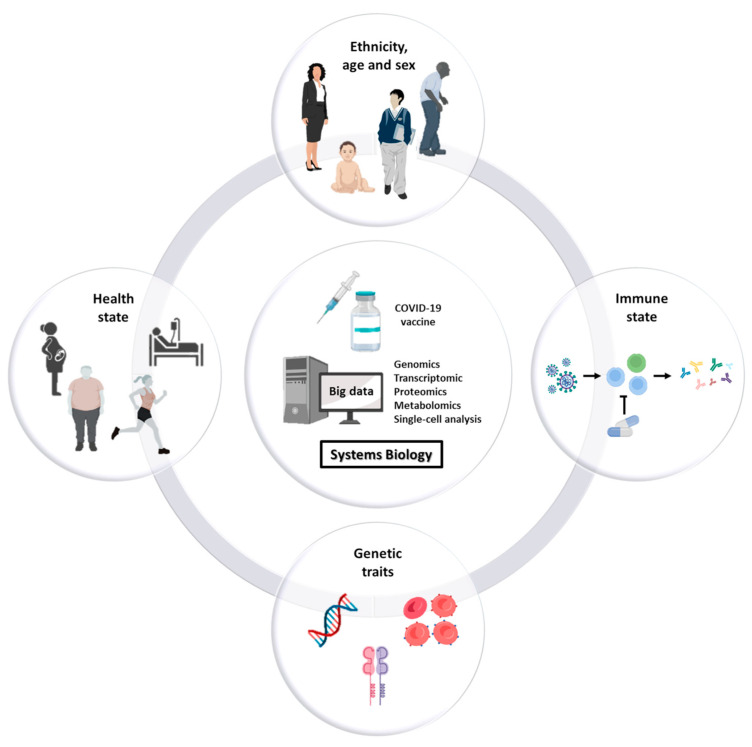
Factors influencing the efficacy and safety of vaccines. Studies show that responsiveness as well as potential adverse reactions to vaccines are dependent on several factors including sex, age, ethnicity, comorbidities, immune state, therapies, and the genetic/biological characteristics of the individual. Systems biology is instrumental to differentiate the cohorts of subjects that will benefit from vaccination from the ones that will not and may develop serious adverse effects after vaccination.

The goal of personalized vaccination is to immunize and protect individuals that are vulnerable to infection, that are responsive to the vaccine and that at the same time are unlikely to develop serious adverse events in the short and long term. High-throughput omics technologies and systems biology for computing big data are instrumental to the identification of the cohort of individuals needing vaccination with a high benefit/risk ratio, taking into account all the factors influencing the efficacy (and safety) of the vaccine, namely sex, age (newborn/infant/adults/elders), immune state (e.g., immunodeficient or immunocompromised vs. immunocompetent; naturally immunized after infection), health state (presence of co-morbidities), immunosuppressive therapies, genetic traits, and others (Figure 1).

For instance, the genetic/biological reason for the lower antibody response after two doses of mRNA COVID-19 vaccine in pregnant compared with non-pregnant women [33] should be investigated at omics level, as should the reason for the rapid and substantial decline in the effectiveness of the vaccine in the general population [10,11,12] and particularly in elders and subjects with a comorbidity [34].

Genome-wide association and epigenome-wide association studies have begun to offer new insights into patients with COVID-19 who are particularly prone to developing severe interstitial pneumonia [35,36,37], while exome and genome sequencing have helped identify a rare variant of TLR7 that may lead to severe COVID-19 outcomes [38]. Genome-wide association studies (GWASs) in COVID-19 patients revealed that risk of intubation and respiratory failure was linked to two critical loci of interest, namely 9q34.2 and 3p21.31; the former locus was associated with the AB0 blood group (and it was found that group A is more susceptible while group 0 is less susceptible) and in the latter locus at least six genes that could play a role in infection and immune response have been defined (*SLC6A20*, *LZTFL1*, *CCR9*, *FYCO1*, *CXCR6*, and *XCR1*) [35]. Specifically, *SLC6A20* encodes sodium-proline transporter 1, which can interact with ACE2; *CCR9*, *XCR1* and *CXCR6* encode chemokine receptors; and *FYCO1* is involved in autophagy (a vesicular process involved in viral assembly and exit). Likewise, exome and genome sequencing revealed that patients with life-threatening COVID-19 pneumonia presented genetic defects at the TLR3 and IRF loci, which are involved in double-stranded RNA sensing and type I IFN immunity [39]. Similarly, GWASs may be useful for identifying the genetic determinants of innate resistance to infection [40]. For instance, prior infections with other coronaviruses may contribute to resistance to SARS-CoV-2 infection due to cross-reactive T-cell-mediated immunity [41], which could be detected by immunocellomics. Immunocellomics and single-cell RNA sequencing were used to determine the causes of an impaired anti-Spike response in tri-vaccinated elderly individuals [42]. The study revealed an enrichment of circulating “atypical” B lymphocytes along with a genetic signature of T-cell exhaustion [42].

Another category of subjects who can benefit from clinical omics profiling is that of immunodepressed (because of concomitant therapies) and immunodeficient patients who will likely not respond to standard vaccination protocols [43,44]. Accordingly, a personalized vaccination strategy has been proposed for rituximab-treated patients in which antibody production is suppressed [45]. Such immunocompromised patients or those on immunosuppressant therapy are given multiple doses to boost and maintain a sufficient level of immunoprotection [44,46]. However, one must then consider the risks of even serious adverse events in the short- and long-term that may become apparent, especially in some susceptible individuals, because of the continuous stresses on the immune system caused by multiple dose vaccinations [47]. In fact, COVID-19 vaccination outcomes demand long-term prospective studies, another area where vaccinomics and adversomics scholarship stands to benefit.

Vaccinomics and adversomics go hand in hand with multi-omics technologies across the biological hierarchy of genomics, proteomics, and metabolomics, not to mention epigenomics. Thus, going forward and contingent on the establishment of a robust evidentiary base in the future, it is likely that one can make use of omics biotechnologies to profile genomic and epigenomic biomarkers associated with the risk of re-infection or of developing adverse events following COVID-19 vaccination. As an example, immunogenomics offers prospects for the identification of MHC-II-related predisposition to re-infection in COVID-19 vaccinated individuals [48]. Similarly, immunocytomics and immunogenomics may identify genes related to T- and NK cell exhaustion and suppression that are associated with adverse reactions to COVID-19 mRNA vaccines [49]. In this context, single-cell mRNA sequencing (scRNA-seq) of peripheral blood lymphocytes and monocytes before and 28 days after vaccination helped to stratify patients with pre-existing clinical disorders that may contribute to adverse reaction to the vaccine [47]. Vaccine-induced thrombosis associated with thrombocytopenia (VITT) has been reported after COVID-19 vaccination, particularly with an adenoviral vector [50], while deep vein thrombosis (DVT) has been reported after lipid nanoparticle-mRNA-based vaccines [51]. The genetic basis of VITT, which though rare is more frequently observed in young females, shares many similarities with SARS-CoV-2-induced coagulopathies, yet it presents unique interactomes associated with the AURKA, CD46 and CD19 genes [52]. Genomic and serological signatures could help identify subjects who are more likely to develop an allergic reaction to specific vaccine components [53,54] or who have a genetic risk of developing venous thromboembolism [55], which may inform and guide preventive interventions.

Many of the reported post-vaccination adverse events are immune-mediated, and it has been hypothesized that certain anti-idiotypes specific to the anti-S primary antibody mimic the original antigen in their potential to produce similar pathological effects in the long-term [56,57]. Omics technologies could be useful for determining the presence and characteristics of the idiotypes of the antibodies induced by the vaccine, and thus help to identify patients potentially at risk of developing adverse effects long after vaccination.

Even more relevant and timely is the possibility offered by the multi-omics technologies in combination with other serological and clinical parameters to profile those individuals for which vaccination would be more beneficial, while excluding those who would not benefit because they are already protected and not particularly susceptible to developing the disease in a severe form and who would be easily treated with available therapies [58]. For instance, immunocytomics could help determine the pool of T and B memory cells that specifically react to common epitopes of previous infection or vaccination and potentially provide protection against a family of viruses sharing those epitopes. These infection-recovered subjects can be further evaluated in light of vaccinomics guidance to determine the most optimal health interventions to prevent or minimize adverse effects [22]. It has been shown that infection-recovered subjects show similar or better protection from COVID-19 than vaccinees in terms of risk of re-infection [58,59,60,61,62], and therefore could be exempted from vaccination. Diverse serious side effects of vaccination in previously naturally immunized subjects have been reported. For instance, myopericarditis was observed following the first dose of the mRNA-COVID-19 vaccine in a patient recovered from mild COVID-19 three months before vaccination [63].

Although antibody-dependent enhancement (ADE) of viral infection seems to be unlikely at the population level [64], it is advisable to exempt individuals possessing anti-COVID-19 antibodies from vaccination according to the precaution principle [65,66,67].

It is now possible to profile immunized (post-infection and post-vaccination) subjects by rapidly and efficiently testing the level and specificity of anti-SARS-CoV-2 antibodies with microfluidic technology [68] and multiplex assays [69].

**Figure 2:**
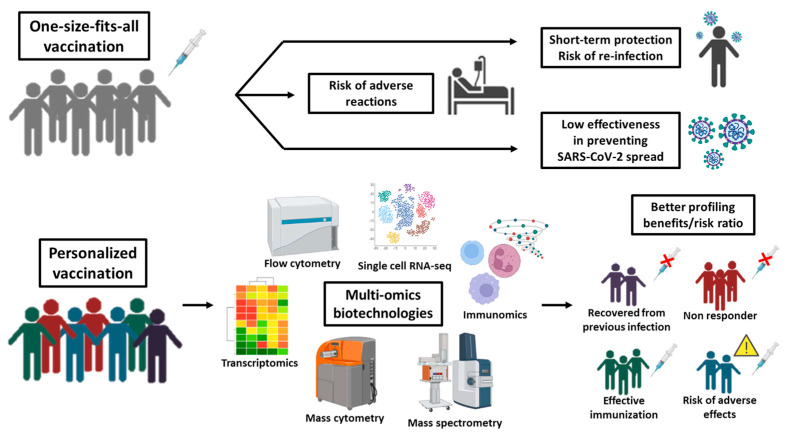
Mass versus personalized vaccination in the omics era. When the vaccine is administered to a large population without considering the individual pathophysiological condition(s), it is highly probable to observe inefficacy and/or immediate/late adverse effects in part of the vaccinated. Omics-biotechnologies allow a better subject profiling by investigating all the variables influencing the efficacy and safety of vaccines in view of personalized vaccination that targets the individuals who can benefit of it with negligible side effects while excluding those who may not respond (because immunocompromised) or are already immunized (by prior infection) or are predisposed to severe reactions (e.g., allergy).

Overall, emerging studies suggest how we could harness multi-omics knowledge to narrow the pool of patients who are most likely to benefit from vaccines while also alerting to the likelihood of adverse events (Figure 2). Going forward, precision/personalized vaccines developed by vaccinomics and adversomics would allow for a more accurate assessment of the benefit/risk ratio of vaccination. This is a formidable opportunity for improving the safety profile of vaccination programs in vulnerable populations and especially in children and adolescents [70].

Thus, it is desirable that the necessary investments in omics biotechnologies be made in the immediate future to exploit their full potential to develop personalized antivirals and vaccines for COVID-19 that would also help future pandemic-preparedness [71,72,73,74].

## 6. Conclusions and Perspectives

Although anti-COVID-19 mass vaccination has helped to control the pandemic in terms of reduced severity of the disease, these vaccines were found to be ineffective in preventing the spread of the virus [75]. Mass vaccination may have different unpredictable outcomes that include the risk of serious immediate or late adverse events. This calls for proactive surveillance and registration of post-vaccination adverse effects [76]. This is particularly true for children, who are subjected to several vaccines in a relatively short period. Particularly, it is questionable whether mass vaccination of children with mRNA-based COVID-19 vaccines is an appropriate approach [77,78].

A personalized approach based on the use of omics technologies allows for categorization of the subjects that are more likely to benefit from vaccination, excluding the ones who may not need it (because they are protected) or who may not respond (because of immunologic impairment), and at the same time helps identify the subjects that could develop adverse effects in the short or long term after vaccination. For instance, scRNA-seq of peripheral mononuclear cells in vaccinated individuals revealed pathophysiological changes in coagulation profiles, renal function, and glucose metabolism along with increased NF-κB signaling and reduced type I interferon responses, suggesting caution in vaccinating frail patients with renal dysfunction, diabetes, and coagulation disorders [47]. Compared to males, females have shown a more vigorous immune response to vaccines and may require a lower dose of the vaccine to minimize adverse effects [24]. ADE reactions could develop after vaccination of immune subjects, which calls for determination of the cut-off for circulating microbe-specific antibodies [79]. Although rare, COVID-19 mRNA vaccines have been associated with several other adverse effects, including myocarditis and pericarditis, Guillain–Barré syndrome, Bell’s palsy, and stroke, among others. These clinical manifestations are likely linked to the ectopic expression of the modified (open state) Spike protein and/or of the lipid nanoparticle carrier [80]. Thus, alternative vaccine formulations might be safer [81]. Exuberant immune and inflammatory responses resulting in autoimmune and systemic reactions may be linked to specific HLA molecules to which the vaccine epitope binds or to specific polymorphisms in genes regulating the immune-inflammatory response [12].

To learn more in view of a personalized vaccinology, it is desirable that DNA, RNA, non-coding RNA, and protein samples from the blood and tissues of vaccinated (with and without adverse effects) and unvaccinated donors are collected and stored in biobanks and made available for omics and systems biology analyses. These studies will help elucidate the mechanisms of the vaccine adverse effects and could be exploited for the design of new vaccines or alternative strategies that minimize or avoid such events.

Vaccines must be safer than the disease they are supposed to prevent because vaccines are given to a large population of otherwise healthy people. Such a vision of planetary health and pandemic prevention also calls for equity in access to precision vaccines and diagnostics.

## Data Availability

Not applicable.
